# The building blocks of phage therapy: from lytic phage identification to preclinical validation against antimicrobial-resistant critical priority bacteria

**DOI:** 10.1128/spectrum.00507-26

**Published:** 2026-05-04

**Authors:** Filipa F. Vale

**Affiliations:** 1 BioISI - Instituto de Biossistemas e Ciências Integrativas, Faculdade de Ciências, Universidade de Lisboa 37809, Lisboa, Portugal; Universidad Tecnica de Ambato, Ambato, Ecuador

**Keywords:** bacteriophage therapy, antibiotic resistance, *Enterobacter*, *Enterobacter cloacae* complex

## Abstract

The fight against antimicrobial resistance is bringing back phage therapy, consisting of the use of lytic phages against specific pathogenic bacteria. For this purpose, libraries of well-characterized lytic phages are crucial, as the phage–bacteria arms race can limit efficacy, making phage cocktails generally more effective. In a recent study by H. -Y. Kuo, C. J. B. Bregente, T. T. D. Thuy, J. H. Hidrosollo, et al. (Microbiol Spectr 13:e00835-25, 2025, https://doi.org/10.1128/spectrum.00835-25), 12 novel lytic phages were isolated against carbapenem-resistant *Enterobacter cloacae* complex, a WHO critical-priority pathogen. These phages showed antibacterial activity *in vitro* and a broad host range across a panel of 80 CR-ECC isolates, as well as improved survival in the invertebrate model *Galleria mellonella*. Testing the two most promising phages in a mouse model showed that one significantly enhanced survival, demonstrating its therapeutic potential. This study offers a roadmap for isolating, characterizing, and evaluating phages for therapeutic use.

## COMMENTARY

Antimicrobial resistance (AMR) is a major global health problem and a One Health challenge, spanning antimicrobial use in humans, animals, and agriculture. Furthermore, climate change can be a driver of AMR spread. Human antimicrobial consumption has risen substantially over the past two decades and, if current trends continue, total global antimicrobial use is projected to increase by roughly 50% by 2030, further intensifying selection pressure for resistance (1, 2). To address this challenge, the WHO developed and periodically updates the Bacterial Priority Pathogens List, a global framework that prioritizes antimicrobial-resistant bacteria for research and development of novel therapeutics (3). Among the critical-priority pathogens is carbapenem-resistant *Enterobacter cloacae* complex (CR-ECC), which has emerged as a major multidrug-resistant and carbapenem-resistant *Enterobacterales* globally (4). CR-ECC are nosocomial opportunistic pathogens that cause pneumonia, urinary tract infections, and sepsis and have limited therapeutic options.

In this context, phage therapy has regained importance and consists of the use of lytic bacteriophages (phages) to specifically lyse target antimicrobial-resistant pathogenic bacteria. It is important to use strictly lytic phages, which kill bacteria without integrating into their genomes, making them safer and more reliable than temperate phages (5). Although safer, lytic phages can still mediate generalized transduction, estimated to represent 1% of total phage particles (6), which, although rare, may contribute to the transfer of virulence or antimicrobial resistance genes with a probability of ~10^−8^ (7). Although rare, it should not be dismissed, underscoring the need to consider generalized transduction as a potential risk during phage therapy. The issue of bacterial resistance to phages should also be considered because of the ongoing phage-bacterium arms race, in which bacteria employ innate and adaptive anti-phage defense systems, while phages evolve a diversity of counter-defense strategies to overcome them (8). A strategy to reduce bacteria’s defense mechanisms is the use of cocktails of genetically diverse strictly lytic phages targeting different receptors or resistance mutants, thereby broadening host range and reducing the likelihood of resistance emergence. However, while broadening host range and limiting receptor-specific resistance, phage cocktails may not fully overcome bacterial defense systems, so resistance emergence remains possible (9). Thus, continued discovery, classification, and characterization of phages are paramount to building comprehensive phage libraries capable of addressing AMR and enabling the development of effective phage cocktails against high-priority pathogens such as the CR-ECC.

In their study, Kuo et al. (10) collected environmental and sewage samples from Taiwan for phage isolation. The collected 15 water samples enabled the isolation of 18 phages forming plaques on an MDR CR-ECC isolate, evidencing a permissive host. To ensure genetic diversity among the newly isolated phages, RAPD fingerprinting analysis was performed, and 12 phages displaying distinct patterns were identified. The isolated phages reached titers of 10⁸–10⁹ PFU/mL, which are compatible with therapeutic use, as high phage titers are essential for downstream purification, formulation, and achieving effective bacterial killing. The double-stranded DNA genomes ranged from 178 to 181 kb and contained 271 to 301 ORFs, without any detectable virulence or antimicrobial resistance genes. The phylogenetic analysis showed that these phages formed monophyletic groups, suggesting novel species of the genus *Pseudotevenvirus* (family *Straboviridae*).

To understand the relevance of phages, it is important that they can infect most of the isolates of the target bacterium. Indeed, the 12 phages show this ability, being able to replicate in 65%–93.75% of the 80 clinical CR-ECC strains tested. Additionally, administration of each phage improved survival in *Galleria mellonella*, an invertebrate model, following infection with a CR-ECC isolate. From the host range and *G. mellonella* results, two phages were selected for murine testing. One phage (CYPEBC012) was chosen for its broad host range, and the other (CYPEBC011) because it uniquely lysed a hypervirulent isolate.

In the murine bacteremia model, CYPEBC012 treatment led to a survival comparable to the antimicrobial-susceptible control, as all mice survived through day 3, and 80% remained alive at day 7. In contrast, CYPEBC011 offered strong early protection, with full survival at day 2, but its effect diminished over time, leaving only 20% of mice alive by day 7. Moreover, after treatment, mice presented reduced bacterial loads in blood and liver, and high phage titers. Collectively, the experimental design of this study demonstrates a stepwise narrowing from a larger initial pool of isolated phages to the selection of a single candidate, CYPEBC012, as the most promising for phage therapy against CR-ECC infections.

Notably, none of the tested phages were effective against all CR-ECC isolates, highlighting the need for using phages in combination either with other phages with complementary host ranges or combined with other antimicrobial agents. The authors also noted other limitations, including the potential for phage resistance, testing only single-dose treatments, and evaluating just two phages in mice. While the benefits of phage cocktails for broadening coverage, enhancing efficacy, or limiting resistance remain to be directly demonstrated in this study, Kuo et al.’s (10) findings underscore the importance of developing phage libraries to support combinatorial testing. Future studies should explore multi-dose regimens, phage cocktails, and combinations with other antimicrobials to optimize therapeutic efficacy and limit resistance.

Overall, this study identifies a novel phage with potential for therapy against antimicrobial-resistant infections, addressing a critical need for new strategies to combat AMR. The findings of Kuo et al. contribute to the development of phage libraries against CR-ECC and illustrate a methodological approach that can be applied to other clinically important AMR pathogens.
